# Chronic Voluntary Alcohol Drinking Causes Anxiety-like Behavior, Thiamine Deficiency, and Brain Damage of Female Crossed High Alcohol Preferring Mice

**DOI:** 10.3389/fphar.2021.614396

**Published:** 2021-03-09

**Authors:** Hong Xu, Hui Li, Dexiang Liu, Wen Wen, Mei Xu, Jacqueline A. Frank, Jing Chen, Haining Zhu, Nicholas J. Grahame, Jia Luo

**Affiliations:** ^1^ Department of Pharmacology and Nutritional Sciences, University of Kentucky College of Medicine, Lexington, KY, United States; ^2^ Department of Pathology, University of Iowa Carver College of Medicine, Iowa City, IA, United States; ^3^ Department of Medical Psychology, Shandong University School of Medicine, Jinan, China; ^4^ Department of Molecular and Cellular Biochemistry, University of Kentucky College of Medicine, Lexington, KY, United States; ^5^ Department of Psychology, Indiana University-Purdue University Indianapolis, Indianapolis, IN, United States; ^6^ Iowa City VA Health Care System, Iowa City, IA, United States

**Keywords:** alcohol use disorder, endoplasmic reticulum stress, oxidative stress, neurodegeneration, neuroinflammation

## Abstract

The central nervous system is vulnerable to chronic alcohol abuse, and alcohol dependence is a chronically relapsing disorder which causes a variety of physical and mental disorders. Appropriate animal models are important for investigating the underlying cellular and molecular mechanisms. The crossed High Alcohol Preferring mice prefer alcohol to water when given free access. In the present study, we used female cHAP mice as a model of chronic voluntary drinking to evaluate the effects of alcohol on neurobehavioral and neuropathological changes. The female cHAP mice had free-choice access to 10% ethanol and water, while control mice had access to water alone at the age of 60-day-old. The mice were exposed to alcohol for 7 months then subjected to neurobehavioral tests including open field (OF), elevated plus maze (EPM), and Morris water maze (MWM). Results from OF and EPM tests suggested that chronic voluntary drinking caused anxiety-like behaviors. After behavior tests, mice were sacrificed, and brain tissues were processed for biochemical analyses. Alcohol altered the levels of several neurotransmitters and neurotrophic factors in the brain including gamma-Aminobutyric acid (GABA), corticotropin-releasing factor, cAMP response element-binding protein (CREB) and brain-derived neurotrophic factor. Alcohol increased the expression of neuroinflammation markers including interleukin-6 (IL-6), tumor necrosis factor alpha (TNF-α), monocyte chemoattractant protein-1 (MCP-1) and C-C chemokine receptor 2 (CCR2). Alcohol also induced cleaved caspase-3 and glial fibrillary acidic protein, indicative of neurodegeneration and gliosis. In addition, alcohol inhibited the expression of thiamine transporters in the brain and reduced thiamine levels in the blood. Alcohol also caused oxidative stress and endoplasmic reticulum (ER) stress, and stimulated neurogenesis.

## Introduction

Alcohol use disorder (AUD) is a chronically relapsing disorder characterized by compulsive alcohol consumption that can cause a variety of physical and mental problems (Grant et al., 2004; Bouchery et al., 2011). It is one of the most prevalent mental disorders in the United States with nearly one-third of adults experiencing it at some point in their lifetime (Grant et al., 2004; Grant et al., 2006; Grant et al., 2015). AUD is comorbid with other mental disorders, particularly anxiety disorder (Kushner et al., 2000; Burns and Teesson, 2002; Petry et al., 2005; Schneier et al., 2010). Anxiety is another common psychiatric condition characterized by excessive fear of the situation (McLean and Anderson, 2009; Bandelow and Michaelis, 2015). Clinical studies have shown AUD and anxiety can both act to initiate and reinforce each other (Kushner et al., 2000; Schneier et al., 2010). Many population surveys have consistently shown that AUD is more common in men whereas anxiety is more prevalent in women (Lewis et al., 1996; Kessler et al., 1997; Hasin et al., 2007). However, the national survey on gender-specific comorbidity rates for AUD and anxiety has indicated that alcohol-dependent women may almost double the risk of developing co-occurring anxiety relative to alcohol-dependent men (Kessler et al., 1997). Although much progress has been made in understanding AUD and many treatment options are available, patients with AUD tend to relapse and their recovery is often compromised, which may result from the failure to treat the comorbid anxiety (Randall et al., 2001; Terra et al., 2006; Schneier et al., 2010).

It is important to investigate whether there is a causative role of alcohol-induced brain injury or neurochemical alterations in the anxiety associated with alcohol abuse. Neurotransmitters are chemical molecules that are synthesized and released by neurons to transmit a message by binding to their receptors of target cells across the synapse. Disruption of neurotransmitter systems is associated with alcohol abuse and anxiety. Alcohol affects multiple neurotransmitter systems and neuropeptides in the brain’s reward and stress circuits including gamma-aminobutyric acid (GABA) and corticotropin-releasing factor (CRF) systems. GABA is the primary inhibitory neurotransmitter in the brain. Alcohol can act on the presynaptic neurons to increase GABA release or act on the postsynaptic neurons to enhance the activity of GABA receptor (Banerjee, 2014). GABAergic inhibitory transmission is involved in alcohol drinking behavior (Roberto et al., 2003; Roberto et al., 2004) and anxiety (Kalueff and Nutt, 2007). CRF is a neuropeptide that can function as a neurotransmitter in stress-related behaviors in mood and anxiety disorders (Koob and Thatcher-Britton, 1985; Risbrough and Stein, 2006; Binder and Nemeroff, 2010). CFR signaling in the amygdala has been shown to mediate alcohol-induced GABA release (Bajo et al., 2008). In addition, CRF signaling has been shown to play a role in drinking behavior in alcoholism and is associated with anxiety-like behavior (Zorrilla et al., 2014).

As suggested by clinical studies and research using animal models, alcohol-induced neurodegeneration in multiple brain regions may underlie neurocognitive deficits in AUD (Harper, 1998; Crews, 2008; Kamal et al., 2020). However, the cellular and molecular mechanisms underlying alcohol-induced neurodegeneration remains unclear. There are several proposed mechanisms for alcohol-induced neurodegeneration. Oxidative stress has been implicated as a key mechanism underlying alcohol-induced neurodegeneration (Chen and Luo, 2010; Yang and Luo, 2015). Oxidative stress is defined as the imbalance between the reactive oxygen species (ROS) and antioxidants. Moreover, oxidative stress is associated with alcohol-induced anxiety-like behavior (Brocardo et al., 2012). Endoplasmic reticulum (ER) stress is another potential mechanism for alcohol-induced brain damage in both developing and adult brains (Yang and Luo, 2015). In addition, neuroinflammation is often associated with neurodegeneration and has been implicated in the pathophysiology of AUD (Hurley and Tizabi, 2013; Ramesh et al., 2013; Kempuraj et al., 2016; Coppens et al., 2019; Erickson et al., 2019). It is therefore important to establish appropriate animal models to elucidate these potential mechanisms.

Mice that are willing to voluntarily drink alcohol over an extended period, achieving pharmacologically relevant blood alcohol concentrations (BACs), may model chronic alcohol abuse in humans. The selectively bred crossed High Alcohol Preferring (cHAP) mice, bred for high ethanol intake when 10% ethanol and water are concurrently available for a month, have been shown to voluntarily consume high amounts of alcohol and demonstrate relatively high BACs that are comparable to those observed in alcohol-dependent humans (Matson and Grahame, 2013; Matson et al., 2014; Xu et al., 2019). We have previously used male cHAP mice to determine the effect of chronic voluntary alcohol on neurochemical alterations and thiamine contents in the brain. We demonstrated that chronic voluntary alcohol exposure increased oxidative stress, endoplasmic reticulum (ER) stress and neuronal apoptosis, and decreased thiamine levels in the brain of male cHAP mice. In the present study, we sought to determine the neurobehavioral and biochemical consequences of chronic alcohol voluntary exposure in female cHAP mice. For the comparison to male cHAP, we also investigated the effects of alcohol on neurochemical changes and thiamine contents in female cHAP mice.

## Materials and Methods

### Materials

Bromodeoxyuridine (BrdU) was obtained from Thermo Fisher Scientific (Rockford, IL). CREB, GFAP, Anti-cleaved caspase-3 and anti-Ki-67 antibodies were obtained from Cell Signaling Technology (Danvers, MA). Anti-BrdU antibody was obtained from Thermo Fisher Scientific (Waltham, MA). Antibodies directed against BDNF, CCR2, MCP-1, MANF, GPR30, TNFα, doublecortin, and OCT1 were obtained from Abcam (Cambridge, MA). Biotinylated goat anti-rabbit IgG was obtained from Vector Laboratories Inc. (Burlingame, CA). Alexa 488-conjugated goat anti-rabbit IgG was obtained from Invitrogen (Carlsbad, CA). Antibodies directed against CRF, NRG1, ErbB4, Caspase-12, CHOP, XBP-1s, ATF6, DNP, and HNE were obtained from Santa Cruz Biotech (Santa Cruz, CA). Anti-SLC19A2 antibody and 3, 3′-diaminobenzidine (DAB) were obtained from Sigma-Aldrich (St. Louis, MO). Anti-IL-6 and anti-SLC19A3 antibodies were obtained from Proteintech (Rosemont, IL). GABA ELISA kit (gAB; RK00663) was obtained from ABclonal (Woburn, MA). Anti-Iba-1 antibody was obtained from Wako Chemicals United States (Richmond, VA).

### Experimental Design

The female cHAP mice were maintained in a 12:12 reverse light cycle colony room (lights on at 21:00 h and off at 09:00 h) with free access to 10% ethanol (v/v) solution and water during the period of 7-month alcohol drinking. After that, alcohol solution was removed, and animals were switched to standard light-dark cycle (lights on at 07:00 h and off at 21:00 h) for 1 week. The animals were then subjected to various behavioral tests including open field (OF), elevated plus maze (EPM), and Morris water maze (MWM) tests. EPM was conducted a week after OF and MWM was performed 4 days after EPM. After MWM, animals were injected with BrdU for 2 days, and then sacrificed. The and brain tissues and blood were collected and subjected to neurochemical analyses.

### Animals and Alcohol Exposure Method

Female cHAP mice from the 31st generation of selection were obtained from Dr. Nicholas J. Grahame at Indiana University Purdue University Indianapolis (Indianapolis, IN, United States) and housed in the University of Kentucky Medical Center Animal Care Facilities. Two-month-old mice were used because they are comparable to human young adults; alcohol usage during this period more likely develops alcohol dependence (Lundahl et al., 1997; Grant, 1998; Hingson et al., 2006; Nair et al., 2016). Female mice were chosen to model the comorbidity of anxiety and AUD because alcohol-dependent women are more likely to develop anxiety than alcohol-dependent men (Kessler et al., 1997), and to compare to the results obtained from male mice (Xu et al., 2019). Animals were maintained in a reverse light cycle colony room in which lights were in 12:12 reverse light-dark cycle. The dark cycle started at 9:00 am. All mice had ad libitum access to food and water throughout the experiment. All experimental procedures were approved by the Institutional Animal Care and Use Committee (IACUC) at the University of Kentucky and performed following regulations for the Care and Use of Laboratory Animals set forth by the National Institutes of Health (NIH) Guide. Mice were given access to alcohol at 60 days of age. The mice had 24-hour-free-choice access to 10% ethanol (v/v) solution and water. Alcohol and drinking water were provided with 50 ml test tube water bottle (#20872, Arcata Pet Supplies, Arcata, CA) fit with a stainless-steel sipper tube (Model OCT-100, Ancare, Belmont, NY). The water bottles were changed every 2 days with freshly prepared ethanol in drinking water or drinking water. The average intake of alcohol was 10.88 ± 0.20 g/kg/d. Mice were weighed before and after alcohol drinking and there was no significant difference between the control and alcohol drinking group (data not shown). After 7 months of alcohol consumption, mice were tested for behavioral scoring, then sacrificed and the brain tissues were harvested for neurochemical studies. The whole blood was also collected for LC-MS analysis of thiamine concentration.

### Determination of Blood Alcohol Concentrations (BACs)

Blood samples were collected at 26 weeks of free-choice access to alcohol and water. The blood was drawn from the tail veins 4 h after the onset of the dark cycle. The time point was selected because the cHAP mice drink more in the dark and peak BECs were observed in the dark cycle (Matson and Grahame, 2013; Matson et al., 2014). The plasma supernatant was extracted, and BACs were measured using an Analox Alcohol Analyzer (Analox Instruments, Lunenburg, MA).

### Behavior Tests

All behavior tests were conducted one week after the alcohol drinking had ended and they were 4–7 days apart to ensure adequate rest and stress relief of animals. Open field (OF) was the first test and it was conducted in two consecutive days; then Elevated plus maze (EPM) was applied in 1 day; whereas Morris water maze (MWM) was completed in five consecutive days. With the lights on at 07:00 h, off at 21:00 h, OF and EPM were conducted at similar times in the morning between 08:00–12:00 h, whereas MWM was conducted between at 12:00–16:00 h according to the standard protocol of rodent behavior core at United Kingdom.

### Open Field

Open field (OF) test is commonly used to measure the levels of exploration and anxiety-like behavior in response to novel environment in mice (Seibenhener and Wooten, 2015). OF was performed in the Rodent Behavior Core (RBC) at the University of Kentucky following the standard procedure for two consecutive days. Prior to the test, animals were brought into the testing room and placed in cleaning cages for 10 min as habituation period. Each mouse was then placed in a square chamber measured 50 cm (length) × 50 cm (width) × 38 cm height with opaque white walls in a multi-unit open field maze (San Diego Instruments), and its activity was recorded for 15 min using EthoVision XT 8.0 video tracking software (Noldus Information Technology, Leesburg, VA, United States). The distance traveled (cm), movement time (s), velocity (cm/s) and time spent in the center zone (s) were recorded and automatically calculated. Center zone is defined as the central area of the chamber measured 25 cm (length) × 25 cm (width). Distance traveled and movement speeds are measures of locomotor activity, whereas the time spent in the center zone is a measure of anxiety in mice.

### Elevated Plus Maze

Elevated plus maze (EPM) is primarily used to assess anxiety-related behavior in rodents (Walf and Frye, 2007). It was conducted under low ambient light conditions with white noise to minimize extraneous auditory stimuli. The EPM apparatus consisted of four arms, two without walls, two enclosed with walls, and each arm measured 30 cm (length) × 6 cm (width). Each mouse was tested in a single session of 5 min to explore the maze. The maze was cleaned after each test with Nature’s Miracle enzymatic cleaning solution to remove animal odors. Animal movements were recorded using EthoVision XT 8.0 video tracking software (Noldus Information Technology, Leesburg, VA, United States). The time spent in each arm and the entries into each arm were analyzed.

### Morris Water Maze

The Morris water maze (MWM) is one of most common behavioral tests of spatial learning for rodents (Vorhees and Williams, 2006). The MWM was performed based on a previously published method with some modifications (Xu et al., 2018). Briefly, each mouse was placed in a round plastic tub with a diameter of 108 cm filled with white-painted water at the temperature of 22–23°C to locate and escape onto a submerged platform. Four visible cues were placed at different points around the tub. Each animal was placed at random locations distal to the platform and allowed to swim to find the hidden platform. If the animal failed to locate the platform within 1 min, it would be gently guided to and remained on the platform for 10 s. Each animal was then placed in a warm cage with a heating pad underneath. There were approximately 5 min in between trials. Each mouse was given four daily trials for five consecutive days. Animal movements were recorded and analyzed using EthoVision XT 8.0 video tracking software (Noldus Information Technology, Leesburg, VA, United States).

### Quantification of Thiamine Concentrations in the Whole Blood

The determination of thiamine concentration in the blood was performed based on a previously described method with some modifications (Kato et al., 2015; Xu et al., 2019). Briefly, whole blood (100 μL) was mixed with 100 μL of 1.2 M ice-cold perchloric acid and kept at 0°C for 15 min. The mixture was centrifuged for 30 min at 15,000 g. 150 μL of supernatant was collected and incubated with 150 μL of 0.6 M KOH/1.8 M potassium acetate for neutralization; then the mixture was centrifuged for 30 min at 15,000 g for desalting. The supernatant (250 μL) was mixed with 250 μL of 4% phosphatase acid and hydrolyzed overnight at room temperature. After that, the supernatant (90 μL) was collected and analyzed for thiamine content. A quadruple volume of acetonitrile/methanol (9:1; v/v) containing an internal standard thiamine-D3 was added to the 90 μL supernatant. The mixture was vortexed and centrifuged for 10 min at 12,000 g. The supernatant was transferred to a new vial, dried with SpeedVac until the final volume was 90 μL. An aliquot of the samples was injected into a LC-MS/MS for the analysis of thiamine concentration.

### BrdU Labeling, Immunohistochemistry and Immunofluorescent Staining

BrdU incorporation labels mitotic cells in the S phase and is used to monitor cell proliferation in the brain. After the last behavioral test (MWM), mice received an intraperitoneal injection of BrdU (50 mg/kg) for two consecutive days. Mice were then anesthetized by intraperitoneal injection of ketamine/xylazine (100 mg/kg/10 mg/kg) and perfused with 0.1 M potassium phosphate buffer (pH 7.2), followed by 4% paraformaldehyde in PBS (pH 7.4). The brain tissues were dissected and postfixed in 4% paraformaldehyde for 48 h followed by cryoprotection in 30% sucrose in PBS. Brains were sectioned (sagittal or coronal section) on a sliding microtome (Leica Microsystems, Wetzlar, Germany) at a thickness of 10 μm at an interval of 20 μm. The sections were then mounted onto Superfrost Plus slides (Fisher).

The immunohistochemical (IHC) staining for NGR1, GPR30, CREB, GFAP, Iba-1, BrdU, doublecortin (DCX), Ki67, and cleaved-caspase-3 in the whole brain was performed as previously described (Xu et al., 2019). Neurochemical alterations in some brain regions, such as PFC and hippocampus, were examined and analyzed. Briefly, the mounted sections were incubated in 0.3% H_2_O_2_ in methanol for 30 min at room temperature and then treated with 0.1% Triton X-100 for 10 min in PBS. The sections were washed with PBS three times and then blocked with 1% BSA and 0.01% Triton X-100 for 1 h at room temperature. The sections were incubated with anti-NGR1 (1:200), anti-GPR30 (1:100), anti-CREB (1:200), anti-GFAP (1:200), anti-Iba-1(1:200), anti-cleaved caspase-3 (1:200), anti-BrdU antibody (1:50), anti-DCX (1:1,000), or anti-Ki-67 antibodies (1:400) overnight at 4°C. Negative controls were performed by omitting the primary antibody. After rinsing in PBS, sections were incubated with a biotinylated goat anti-rabbit IgG (1:200) for 1 h at room temperature. The sections were washed 3 times with PBS, then incubated in avidin–biotin–peroxidase complex (1:100 in PBS) for 1 h and developed in 0.05% 3,3′-diaminobenzidine (DAB) containing 0.003% H2O2 in PBS. To quantify positive cells, the positive cells were counted at ×20 magnification from five microscopic fields in the SVZ or DG area from each brain. The average of the positive cells from 4-5 consecutive sections were analyzed using the software of Image lab 5.2 (Bio-Rad Laboratories, Hercules, CA) for each brain.

### Immunoblotting

The brain tissues from the whole brain were frozen in liquid nitrogen and stored at −80°C. Proteins were extracted as previously described (Xu et al., 2019). In brief, the tissues were homogenized using an ice-cold lysis buffer containing 50 mM Tris-HCl (pH 7.5), 150 mM NaCl, 1 mM EGTA, 1 mM PMSF, 0.5% NP-40, 0.25% SDS, 5 μg/ml leupeptin, and 5 μg/ml aprotinin. Homogenates were centrifuged at 20,000 g for 30 min at 4°C and the supernatant fraction was collected. Aliquots of the protein samples (30 μg) were separated on an SDS-polyacrylamide gel by electrophoresis. The separated proteins were transferred to nitrocellulose membranes. The membranes were blocked with either 5% milk or 5% bovine serum albumins (BSA) or at room temperature for 1 h. Subsequently, the membranes were probed with primary antibodies directed against target proteins overnight at 4°C. After three quick washes in TPBS, the membranes were incubated with a secondary antibody conjugated to horseradish peroxidase. The immune complexes were detected by the enhanced chemiluminescence method. The density of immunoblotting was quantified with the software of Quantity One (Bio-Rad Laboratories, Hercules, CA).

### Quantitative Real-Time RT-PCR

Total RNA from the brain was extracted using Trizol Reagent (Life Technologies) and treated with RNA-free DNAase I to remove remnant DNA as described previously (Kim et al., 2014). 1 μg of total RNA was used for first strand cDNA synthesis (Promega, A3500). Quantitative real-time RT-PCR was performed on a Light cycler 480 system (Roche) using a Power SYBR Green PCR Master kit (Invitrogen, 4368706) with cDNA and primers (1 μM) according to the manufacturer’s recommendation. The primers used for this study were purchased from Fisher Scientific and were as follows: TNF-α, Mm099999068; IL-6, Mm00446190. The relative difference between control and treatment group was expressed and calculated as relative increases using 2-∆∆Ct and setting control as 1.

### Measurement of Estradiol, Progesterone and GABA

General Gamma-Aminobutyric Acid ELISA Kit was used to measure GABA concentrations in the homogenized brain tissues. The brain tissues were homogenized in an ice-cold lysis buffer containing 50 mM Tris-HCl (pH 7.5), 150 mM NaCl, 1 mM EGTA, 1 mM PMSF, 0.5% NP-40, 0.25% SDS, 5 μg/ml leupeptin, and 5 μg/ml aprotinin. Homogenates were centrifuged at 20,000 g for 30 min at 4°C and the supernatant fraction was collected. After 100-fold dilution, the optical density of glutamate and GABA was measured at 450 nm using Beckman Coulter DTX880 Multimode Detector according to the manufacturer’s protocol. The concentrations of GABA were calculated based on a standard optical density.

The measurement of estradiol and progesterone in blood plasma was performed by the Ligand Assay and Analysis Core in Center for Research in Reproduction (CRR) at University of Virginia. The plasma in mouse blood was collected following the standard protocol published by CRR. Briefly, whole mouse blood was collected with anticoagulant. The blood sample was then centrifuged at 2000 × g for 15 min at room temperature. The plasma was then transferred into a polypropylene tube and stored at −20°C until shipment for analysis.

### Statistical Analysis

All values were reported as mean ± SEM. A statistical analysis was performed using Graphpad Prism 6 (San Diego, CA, United States) and SPSS software 19 (IBM, Armonk, NY, YSA). *t*-test was used for the data analysis of and EPM, protein markers and molecules between control and alcohol groups. ANOVA was performed to compare difference between treatments with repeated measure factors being day. The Greenhouse–Geisser correction was used to correct the F statistic and assess significance if necessary. Differences were considered significant if the *p* value was smaller than 0.05.

## Results

### Chronic Voluntary Alcohol Exposure Causes Anxiety-like Behaviors in Female cHAP Mice

The animals were weighed before alcohol drinking and 3.5 months after alcohol drinking. The mean body weight of the control and the alcohol drinking group before alcohol drinking was 22.8 ± 0.4 g and 22.2 ± 0.5 g, respectively. The mean body weight of the control and the alcohol drinking group after 3.5 months of alcohol drinking was 22.2 ± 0.3 g and 23.0 ± 0.5 g, respectively.

**FIGURE 1:**
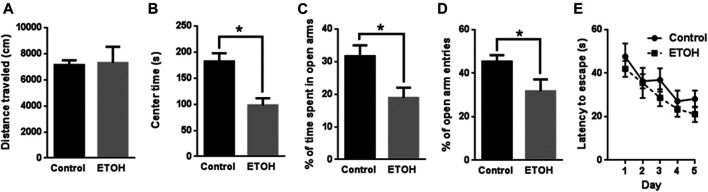
Effects of 7 months of voluntary alcohol drinking on anxiety-like behavior in female cHAP mice. After that, mice were subjected to Open Field (OF) test **(A,B)**, Elevated Plus Maze (EPM) test **(C,D)**, and Morris Water Maze (MWM) test **(E)**. The total distance traveled **(A)** and the time spent in the center **(B)** in OF was measured and presented as the mean ± SEM (n = 7) in each group. The percentage of time spent in open arms **(C)** and percentage of entry numbers into open arms **(D)** in EPM were quantified and presented the mean ± SEM (n = 7). **(E)** The spatial learning and memory were evaluated by MWM. The total latency escape time was measured and presented as the mean ± SEM (n = 7). Unpaired student t-test was used to assess the difference between control and alcohol-exposed group. *p < 0.05 denotes a statistically significant difference from the control group.

The blood alcohol concentrations (BACs) were determined 26 weeks into the alcohol drinking history. The mean BAC was 77.9 ± 8.51 mg/dl. In the open field (OF) test, we measured the total distance traveled and time spent in the center in two consecutive days. Because the data are similar in both days, we averaged the data, so each data point represents the mean of the measurements of 2 days in Figure 1A. The total distance traveled by the control mice was 7,132 ± 368.4 cm (mean ± SEM) (n = 7) which was not significantly different from that by the alcohol-exposed mice (7,294 ± 1,237 cm, n = 7) [t (12) = 0.1255, *p* = 0.902] (Figure 1A). The time spent in the center by the alcohol-exposed mice (98.11 ± 13.46 s, n = 7), however, was significantly lower than that by the control mice (182.0 ± 15.77 s, n = 7) [t (12) = 4.047, *p* = 0.002] (Figure 1B), suggesting anxiety-like behaviors.

We then conducted elevated plus maze (EPM) and measured the time and entry number in open and closed arms. The percentage of time in open arms for the alcohol-exposed mice was measured as 18.90% ± 3.11% (n = 7) which was significantly lower than that for the control mice 31.69% ± 3.31% (n = 7) [t (12) = 2.816, *p* = 0.016] (Figure 1C). The percentage of entry numbers of open arms for the alcohol-exposed mice was 31.75% ± 5.40% (n = 7) which was also significantly lower than that for the control mice 45.33% ± 2.97% (n = 7) [t (12) = 2.202, *p* = 0.048] (Figure 1D). These results indicated anxiety-like behavior in alcohol-exposed mice.

In addition, we applied Morris water maze (MWM) to determine whether spatial learning was affected by alcohol exposure. Animals were trained with four trials per day for 5 days and the time required to locate the hidden platform in water maze were analyzed and presented in Figure 1E. The statistical analysis indicated there was a significant main effect of day [F (4, 48) = 7.294, *p* < 0.001] but not treatment [F (1, 12) = 1.959, *p* = 0.187]. The interaction of day by treatment was not significant [F (4, 48) = 0.226, *p* = 0.922]. Therefore, alcohol exposure did not alter spatial learning.

### Chronic Voluntary Alcohol Exposure Alters the Levels of Neurotransmitters and Neurotrophic Factors That Are Related to the Regulation of Anxiety

**FIGURE 2:**
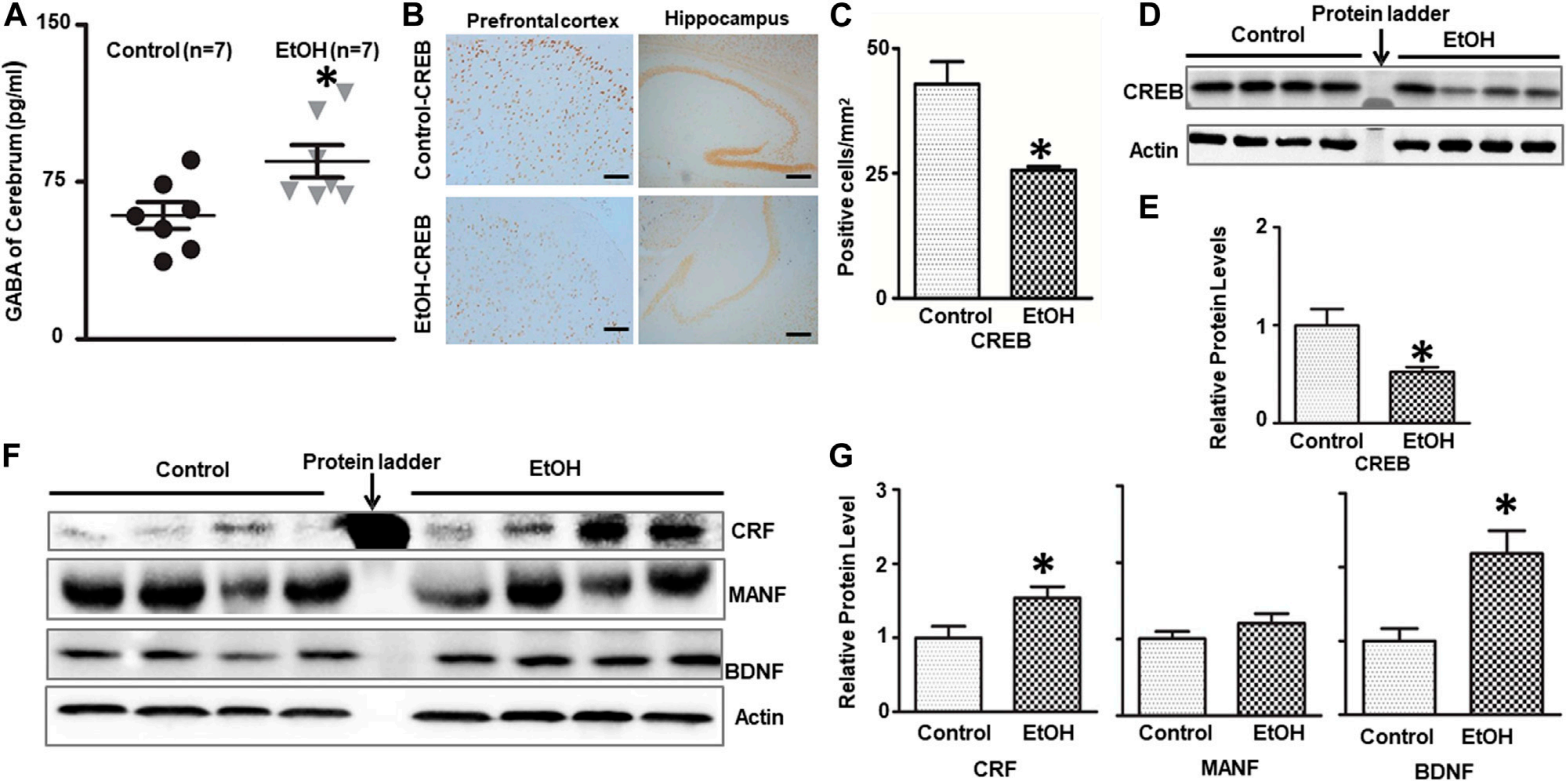
Effects of chronic alcohol exposure on the levels of GABA and neurotrophic factors in the brain. **(A)** The concentration of GABA in the brain was determined by ELISA as described in the *Materials and Methods*. n = 7, *p < 0.05 denotes a statistically significant difference from the control. **(B)** The expression of CREB in the cerebrum was determined by immunohistochemistry (IHC). **(C)** The numbers of CREB-positive cells in the hippocampus or prefrontal cortex (PFC) were determined. The results were expressed as the mean ± SEM; n = 4, *p < 0.05 denotes a statistically significant difference from the control group. **(D)** The expression of CREB was determined by immunoblotting (IB). **(E)** The relative amounts of CREB were quantified and normalized to the expression of actin. **(F)** The expression of CRF, BDNF and MANF in the cerebrum was determined by IB. **(G)** The relative amounts of CRF, BDNF and MANF were quantified and normalized to the expression of actin. The results were expressed as the mean ± SEM, n = 4 for each group. *p < 0.05 denotes a statistically significant difference from the control group.

γ-Aminobutyric acid (GABA) is the major inhibitory neurotransmitter controlling synaptic transmission and neuronal excitability. GABAergic inhibitory transmission is involved in the effects of alcohol exposure on the brain and anxiety behaviors (Roberto et al., 2003; Roberto et al., 2004; Kalueff and Nutt, 2007). We measured the GABA concentration in the brain after 7 months of alcohol exposure. As shown in Figure 2A, alcohol exposure significantly increased the concentration of GABA in the brain; it was 84.82 ± 7.72 (pg/ml) in the alcohol-exposed group (n = 7) and 58.89 ± 6.39 (pg/ml) in the control mice (n = 7) [t (12) = 2.587, *p* = 0.024].

We next examined the expression of several neurotrophic factors/growth factors that are known to play a role in anxiety and alcoholism, such as CRF, CREB, and BDNF (Bolaños and Nestler, 2004; Pandey et al., 2004; Govindarajan et al., 2006; Uddin and Singh, 2007; Baiamonte et al., 2014; Roberto et al., 2017). As shown in Figures 2B–E, chronic alcohol exposure decreased the number of CREB-positive cells in the prefrontal cortex (PFC) and hippocampus [t (6) = 3.758, *p* = 0.0094], and down-regulated the expression of CREB in the brain [t (6) = 2.660, *p* = 0.038]. On the other hand, alcohol exposure increased the levels of CRF [t (6) = 2.481, *p* = 0.048] and BDNF [t (6) = 3.143, *p* = 0.016] in the brain without affecting MANF [t (6) = 1.360, *p* = 0.229], a protein involved in ER function (Figures 2F,G).

### Chronic Voluntary Alcohol Exposure Causes Oxidative Stress, ER Stress and Apoptosis in the Brain

**FIGURE 3:**
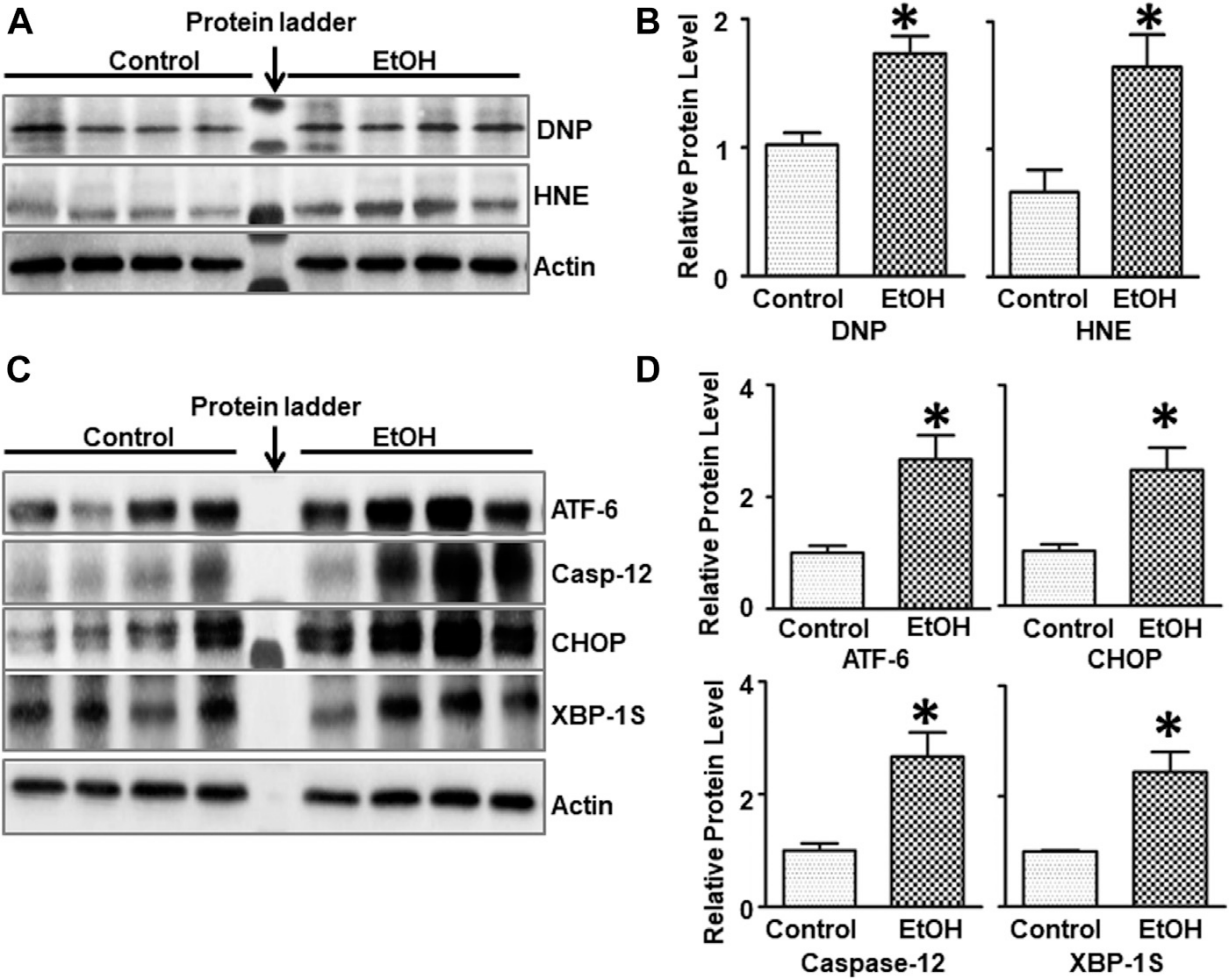
Effects of chronic alcohol exposure on oxidative stress and ER stress in the brain. **(A)** The expression of 4-HNE and DNP in the cerebrum was determined by IB. **(B)** The relative amounts of 4-HNE and DNP were quantified and normalized to the expression of actin. **(C)** The expression of ER stress markers, ATF-6, CHOP, Caspase-12, and XBP-1s in the cerebrum was determined by IB. The relative amounts of expression were quantified and normalized to the expression of actin. **(D)** The relative amounts of ER stress markers were quantified and normalized to the expression of actin. The results were expressed as the mean ± SEM; n = 4 for each group. *p < 0.05 denotes a statistically significant difference from the control group.

Since oxidative stress and ER stress play an important role in alcohol-induced damage to the CNS (Yang and Luo, 2015), we sought to investigate the effect of chronic alcohol exposure on oxidative stress and ER stress in the brain. 4-hydroxynonenal (4-HNE) and 2, 4-dinitrophenol (DNP) are reliable biomarkers for lipid peroxidation and protein oxidation, respectively (Perluigi et al., 2014). As shown in Figures 3A,B, chronic alcohol drinking significantly increased the expression of 4-HNE [t (6) = 2.925, *p* = 0.026], and DNP [t (6) = 3.915, *p* = 0.008] in the brain, indicating the induction of oxidative stress. Furthermore, chronic alcohol drinking upregulated the expression of a number of markers for ER stress, such as ATF-6 [t (6) = 2.822, *p* = 0.030], CHOP [t (6) = 2.718, *p* = 0.035], Caspase-12 [t (6) = 3.341, *p* = 0.01], and XBP-1s [t (6) = 3.016, *p* = 0.024] in the brain, indicative of ER stress (Figures 3C,D).

**FIGURE 4:**
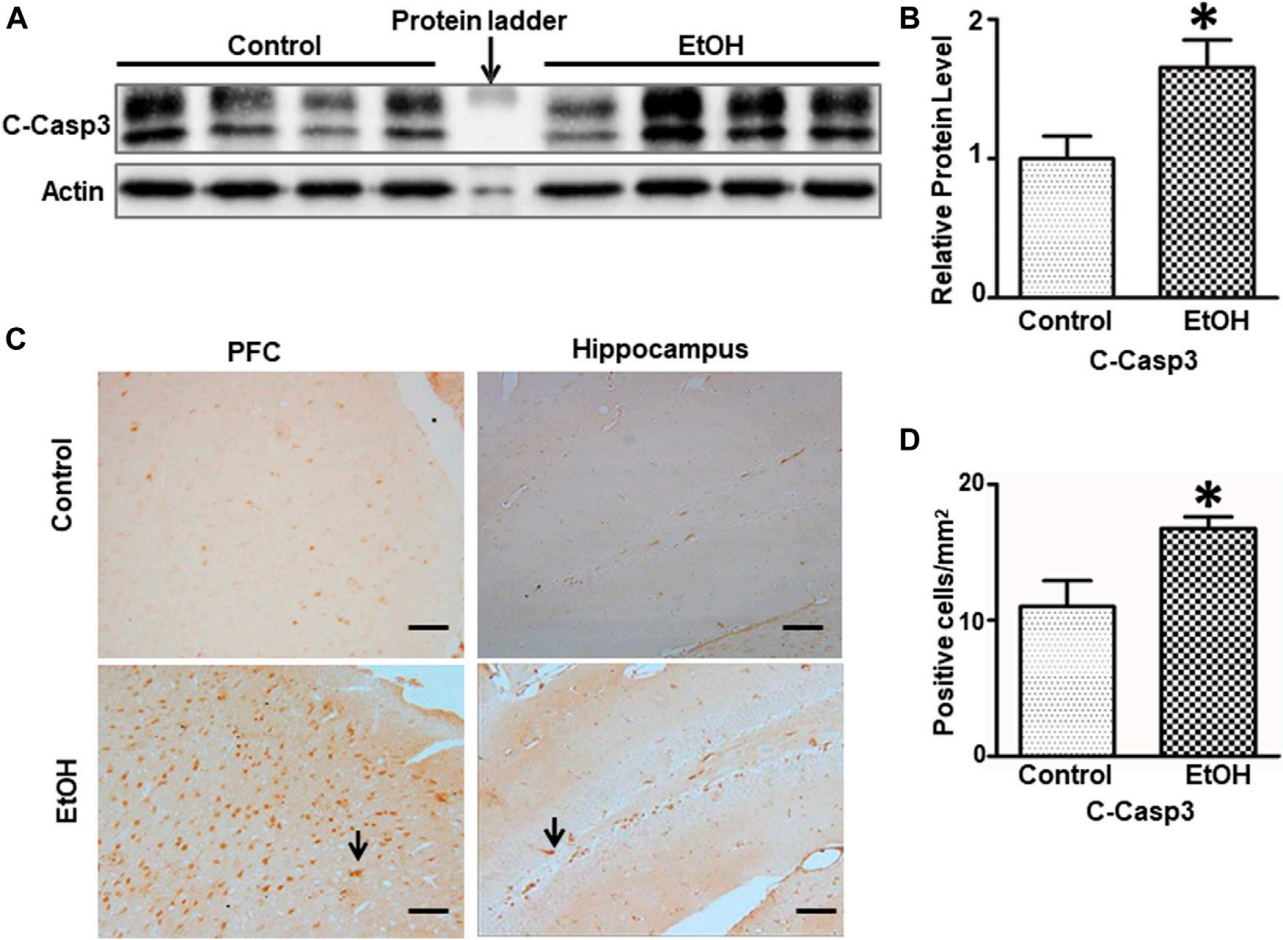
Effects of chronic alcohol exposure on the expression of activated caspase-3. **(A)** The expression of cleaved caspase-3 in the cerebrum was determined by IB. **(B)** The relative amounts of expression were quantified and normalized to the expression of actin. The result was expressed as the mean ± SEM; n = 4. *p < 0.05 denotes a statistically significant difference from the control group. **(C)** The expression of cleaved caspase-3 in the prefrontal cortex (PFC) and the dentate gyrus (DG) of the hippocampus was examined by IHC; bar = 50 μm. Arrows indicate cells that are positive for cleaved caspase-3. **(D)** The numbers of cleaved caspase 3-positive cells in the hippocampus and PFC were determined. The results were expressed as the mean ± SEM; n = 4.

We next sought to determine whether alcohol caused neuronal apoptosis in the brain. As shown in Figures 4A,B, the alcohol-induced increase in cleaved-caspase-3 was shown by immunoblotting analysis [t (6) = 2.567, *p* = 0.043], indicating apoptosis in the brain. Consistently, alcohol increased the expression of cleaved-caspase-3 in the PFC and the dentate gyrus (DG) of hippocampus was revealed by IHC [t (6) = 2.756, *p* = 0.033] (Figures 4C,D).

### Chronic Voluntary Exposure Induces Neuroinflammation and Glial Activation in the Brain

**FIGURE 5:**
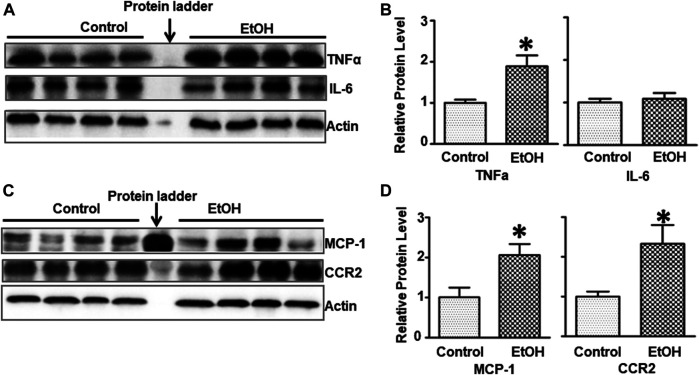
Effects of chronic alcohol exposure on inflammatory cytokines/chemokines in the brain. **(A)** The expression of IL-6 and TNFα in the brain was determined with IB. **(B)** The relative expression of IL-6 and TNFα was quantified as described above. Each data point was the mean ± SEM; n = 4. *p < 0.05, statistically significant difference from the control group. **(C)** The expression of MCP-1 and CCR2 in the brain was determined with IB. **(D)** The relative expression of MCP-1 and CCR2 was quantified. Each data point was the mean ± SEM; n = 4. *p < 0.05, statistically significant difference from control group.

Alcohol-induced damage to the brain is usually accompanied by neuroinflammation (Chastain and Sarkar, 2014; Zhang and Luo, 2019). We sought to determine whether chronic alcohol exposure induced neuroinflammation. Interleukin-6 (IL-6), tumor necrosis factor alpha (TNFα), monocyte chemoattractant protein-1 (MCP-1) and its receptor CCR2 are major pro-inflammatory cytokines/chemokines that are involved alcohol-induced in injuries in the CNS (Vallières and Rivest, 1999; Alfonso-Loeches et al., 2016; Zhang et al., 2018). Alcohol exposure significantly increased the protein levels of TNFα [t (6) = 3.172, *p* = 0.019] but not IL-6 [t (6) = 0.502, *p* = 0.634] in the brain of female cHAP mice (Figures 5A,B). Chronic alcohol drinking also significantly upregulated the expression of MCP-1 [t (6) = 3.039, *p* = 0.023] and CCR2 [t (6) = 2.916, *p* = 0.027] in the brain (Figures 5C,D), indicating the induction of neuroinflammation.

**FIGURE 6:**
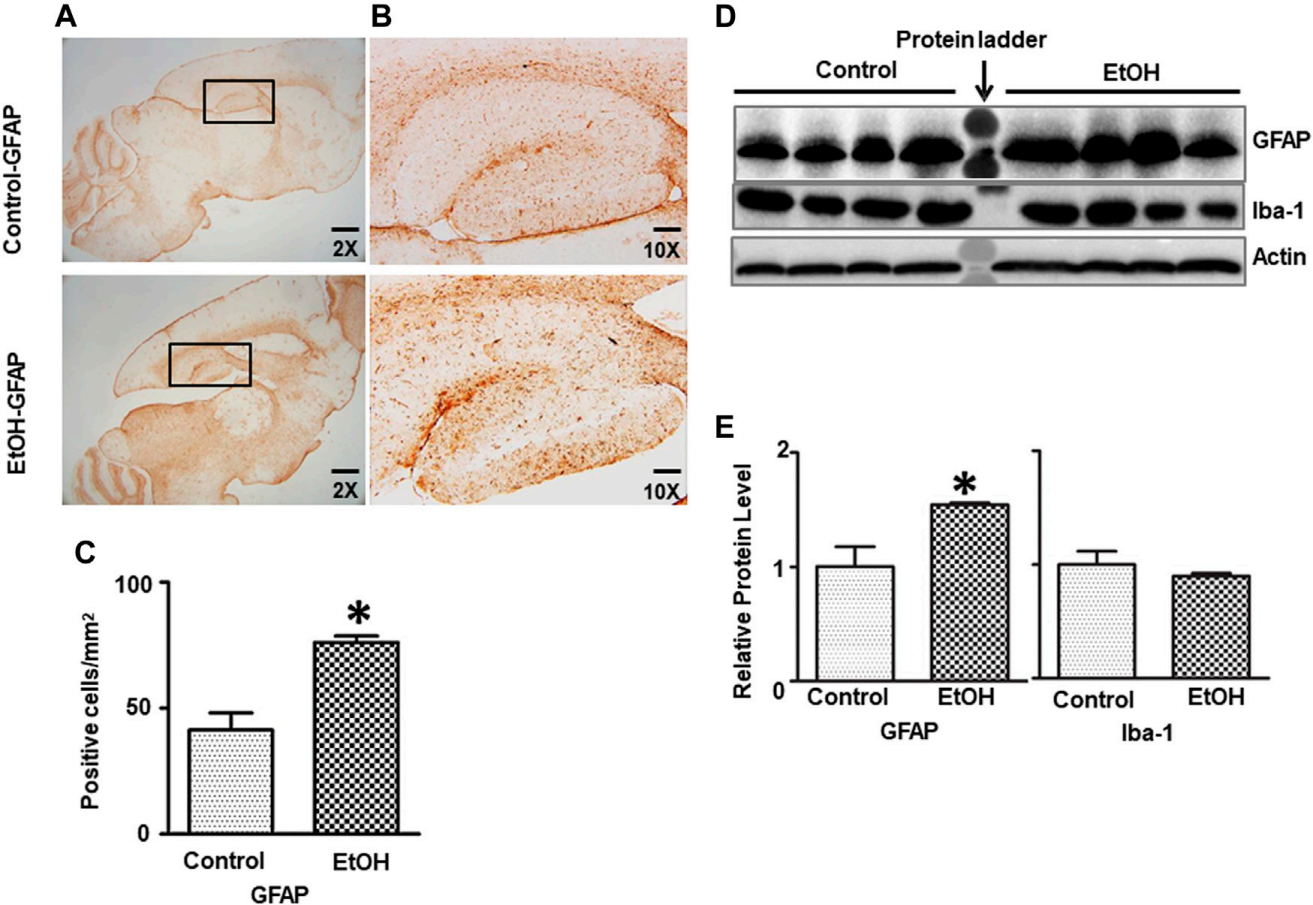
Effects of chronic ethanol exposure on astrocyte activation. **(A)** The expression of GFAP in the mouse brain was examined by IHC. The rectangle insets indicate an area of the hippocampus. **(B)** The rectangle insets in **(A)** are shown in higher magnifications. **(C)** The GFAP-positive cells in the hippocampus were quantified. The result was expressed as the mean ± SEM, n = 3 for each group. *p < 0.05 denotes a statistically significant difference from the control group. **(D)** The protein levels of GFAP were examined by IB. **(E)** The relative amounts of GFAP expression were quantified and normalized to the expression of actin. The result was expressed as the mean ± SEM, n = 4 for each group. *p < 0.05 denotes a statistically significant difference from the control group.

We further determined whether chronic alcohol exposure activated astrocytes or microglia. As shown in Figure 6, the IHC study indicated that GFAP positive cells were increased in the hippocampus of alcohol-exposed mice [t (6) = 4.77, *p* = 0.008] (Figures 6A–C). This was confirmed by immunoblotting analysis which indicated that chronic alcohol exposure increased the expression of GFAP [t (6) = 3.123, *p* = 0.020] (Figures 6D,E), suggesting gliosis in the brain. However, alcohol exposure did not activate microglia and alter Iba1 expression (data not shown).

### Chronic Voluntary Alcohol Exposure Reduces Thiamine Concentration in the Blood, and the Expression of GPR30 in the Brain

**FIGURE 7:**
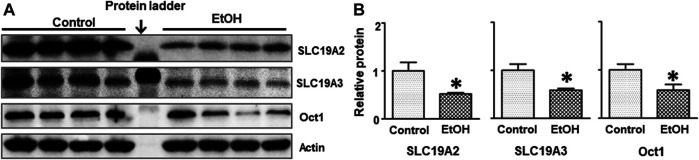
Effects of chronic alcohol exposure on the expression of thiamine transporters in the brain. **(A)** The expression of thiamine transporters (SLC19A2, SLC19A3, and OCT1) in the cerebrum was determined by IB. **(B)** The relative amounts of expression were quantified and normalized to the expression of actin. The results were expressed as the mean ± SEM, n = 4 for each group. *p < 0.05 denotes a statistically significant difference from the control group.

Thiamine deficiency (TD) has long been associated with alcohol-induced brain damages (Leevy and Baker, 1968; Woodhill and Nobile, 1972; Butterworth, 1995). We sought to determine whether alcohol exposure reduced thiamine level in the blood. As shown in Table 1, the concentration of thiamine in alcohol-exposed mice was 0.03 + 0.003 nmol/ml (n = 8), while it was 0.66 ± 0.15 nmol/ml in control animals (n = 8) [t (14) = 4.25, *p* = 0.001]. Alcohol exposure also significantly reduced the expression of thiamine transporters SLC19A2 [t (6) = 2.656, *p* = 0.038], SLC19A3 [t (6) = 3.056, *p* = 0.022], and OCT1 [t (6) = 2.502, *p* = 0.046] in the brain (Figure 7). However, alcohol did not alter these thiamine transporters in the liver (data not shown).

**TABLE 1:** Thiamine concentrations in the whole blood.

Average thiamine concentration (nmol/ml)	Control EtOH
**Mean**	0.66 0.03*
**SEM**	0.15 0.003

Effects of chronic alcohol exposure on thiamine concentrations in the blood. The concentration of thiamine in the blood was determined by LC-MS/MS as described in the “*Materials and Methods*”. The results were expressed as mean ± SEM; *n* = 8 for both control and EtOH group. *denotes a statistical difference, *p* < 0.05.

**FIGURE 8:**
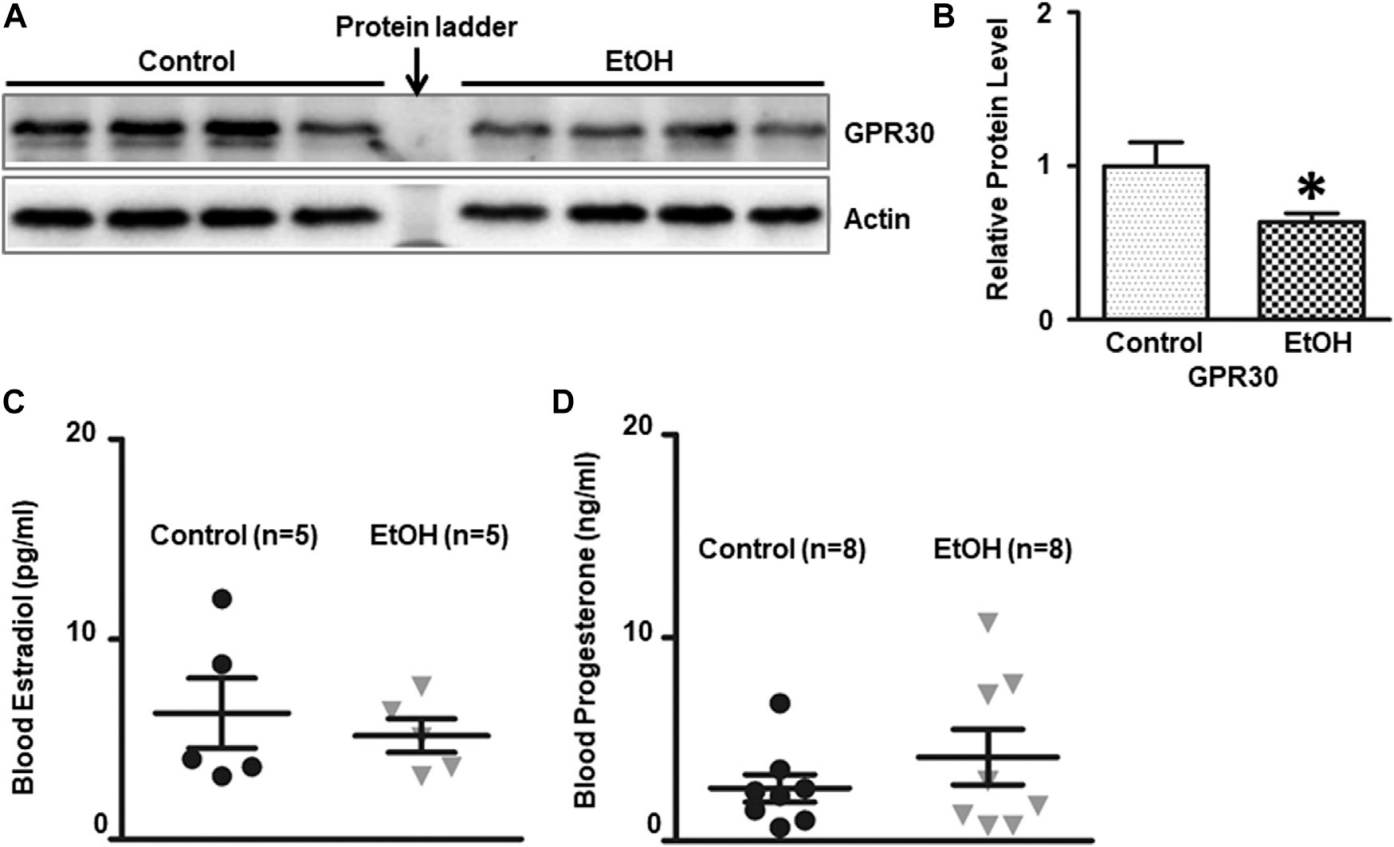
Effects of chronic alcohol exposure on the expression of G-protein-coupled estrogen receptor (GPR30) in the brain, and estradiol/progesterone levels in the blood. **(A)** The expression of GPR30 in the cerebrum was determined by IB. **(B)** The relative amounts of expression were quantified and normalized to the expression of actin. The results were expressed as the mean ± SEM, n = 4 for each group. *p < 0.05 denotes a statistically significant difference from the control group. The concentration of estradiol **(C)** and progesterone **(D)** in the blood was determined by ELISA as described in the *Materials and Methods*. n = 5 for estradiol test, and n = 8 for progesterone test.

There is a gender difference in the neurobiology of anxiety, and females face twice the risk of anxiety disorder as males (Lebron-Milad and Milad, 2012; Cover et al., 2014; Bale and Epperson, 2015), which suggests that sexual hormones may play a role. G-protein-coupled estrogen receptor (ER) known as GPR30 has been implicated in the rodent anxiety response. We determined the expression of GPR30 in the brain. Chronic alcohol exposure significantly decreased the level of GPR30 [t (6) = 2.484, *p* = 0.048] (Figures 8A,B) in the brain. However, there was no significant difference of estradiol and progesterone concentrations in the blood between control and alcohol-exposed mice. The concentration of estradiol in the blood was 6.31 ± 1.75 (pg/ml)(n = 5), and 5.18 ± 0.84 (pg/ml) in alcohol-exposed (n = 5) and control group (n = 5), respectively [t (8) = 0.581, *p* = 0.577] (Figure 8C). The concentration of progesterone in the blood was 4.13 ± 1.36 (ng/ml), and 2.59 ± 0.68 (ng/ml) in alcohol-exposed (n = 8) and control group (n = 8) for respectively [t (14) = 1.01, *p* = 0.329] (Figure 8D).

### Chronic Voluntary Alcohol Exposure Stimulated Neurogenesis in the Brain

**FIGURE 9:**
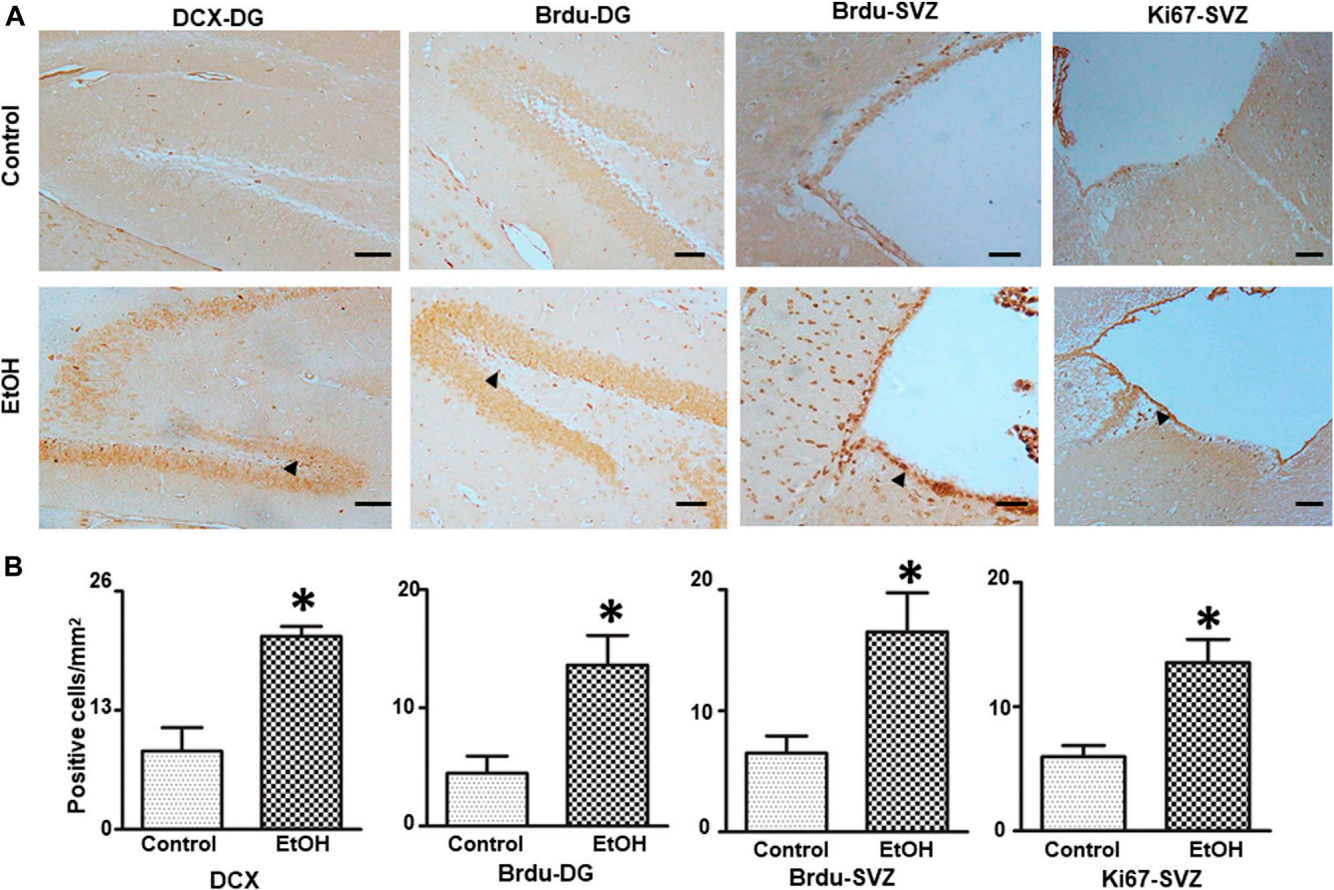
Effects of chronic alcohol exposure on neurogenesis in the subventricular zone (SVZ) and dentate gyrus (DG) of the hippocampus. Both control and alcohol-exposed mice received BrdU injection. **(A)** BrdU-positive cells in the DG and SVZ were determined by IHC. Arrows indicate BrdU-positive cells; bar = 50 μm. The expression of doublecortin (DCX) in the DG of the hippocampus and Ki67 in the SVZ were examined by IHC. Arrows indicate DCX- and Ki67-positive cells; bar = 20 μm. **(B)** The number of DCX-, BrdU-, and Ki67-positive cells in the DG and SVZ was quantified as described in the “*Materials and Methods*” section. The results were expressed as mean ± SEM, *n* = 3 or 4 for each group. *p < 0.05 denotes a statistically significant difference from the control group.

We investigated the effect of chronic alcohol exposure on the neurogenesis in the subventricular zone (SVZ) and the dentate gyrus (DG) of the hippocampus. As shown in Figure 9, alcohol exposure increased the number of BrdU-positive cells [t (4) = 3.16 , *p* = 0.034 ] and DCX-positive cells [t (4) = 4.50 , *p* = 0.011 ] in the DG; Alcohol exposure also increased the number of BrdU-positive cells (t (5) = 3.17 , *p* = 0.025 ] and Ki67-positive cells [t (6) = 3.62 , *p* = 0.01 ] in the SVZ, indicating enhanced proliferation of neural progenitors.

## Discussion

In the present study, we used female cHAP mice to investigate chronic voluntary alcohol drinking-induced neurobehavioral and neurochemical alterations. The mice drank alcohol for 7 months, which is roughly equivalent to 20 years of drinking in humans extrapolated as a proportion of the lifespan. These are the first data showing behavioral symptoms of alcohol withdrawal in these mice, which are quite resistant to more conventional measures of withdrawal, such as handling induced convulsions following multiple rounds of chronic vapor exposure and withdrawal over several weeks (Lopez et al., 2011). Our study suggests that cHAP mice are valuable in their ability to use voluntary drinking to model both behavioral and biochemical effects of chronic alcohol consumption and subsequent short-term withdrawal in human AUD.

### Chronic Voluntary Alcohol Drinking Induces Anxiety-like Behaviors

We conducted three behavioral tests: Open Field (OF), Elevated Plus Maze (EPM) and Morris water maze (MWM) to evaluate the neurobehavioral consequences of 7-month voluntarily alcohol drinking on female cHAP mice (Figure 1). In OF, the female cHAP mice after 7 months of voluntarily alcohol drinking showed no deficits in their locomotor activity but displayed anxiety-like behaviors by spending less time in the center (46.1% reduction). In EPM, the alcohol-exposed animals demonstrated anxiety-like behaviors by spending 40.4% less time and 30.0% less entry number in the open arms. These findings are consistent with a broad range of literature indicating changes in anxiety and stress sensitivity following chronic ethanol exposure (Becker, 2012), but is the first time that any behavioral symptoms of withdrawal have been observe in cHAP mice. In MWM, however, there was no effect of a history of alcohol drinking on latency to escape, suggesting that spatial learning was not affected by alcohol exposure. Previous studies demonstrating decreased MWM learning after alcohol exposure were in rats (Lukoyanov et al., 1999; Novier et al., 2016), and it is unclear why we did not observe these changes in this study, but species may be a factor. Besides species, the age of animals being studied is a potential factor. For example, adolescents are more sensitive to alcohol-induced impairments in the acquisition of spatial learning and memory than adult rats (Acheson et al., 2001). Moreover, the duration of withdrawal may also be a factor. Chronic alcohol consumption for 8 weeks (20% ethanol in liquid diet) followed by a three or 12-weeks of withdrawal period produced significant deficits in learning and memory in male mice (Farr et al., 2005).

Overall, these findings may be of particular interest because comorbidity of AUD and anxiety is more common in women (Kessler et al., 1997) though AUD is more common in men (Lewis et al., 1996; Kessler et al., 1997; Hasin et al., 2007). Given that mice were showing elevated anxiety at least a week following the cessation of voluntary alcohol access, these findings are consistent with the idea that chronic drug self-administration causes homeostatic alterations in reward and stress systems that persist following cessation of drug use (Breese et al., 2005; Koob et al., 2014), and may drive subsequent relapse. In our previous study using male cHAP, we did not evaluate the effects of alcohol exposure on neurobehavioral outcomes, and therefore cannot make a conclusion whether this is a sex-specific effect. Nonetheless, the model of chronic voluntarily alcohol exposure in female cHAP mice could be useful to study the comorbidity of AUD with anxiety.

We examined several molecules/proteins that are known to be alcohol-sensitive and related to the control of anxiety behavior. We found that alcohol exposure increased GABA, CRF and BDNF, while decreasing CREB in the brain (Figure 2). GABAergic inhibitory transmission has been implicated as a contributory factor to the complex relationship between alcoholism and anxiety (Silberman et al., 2009). While some previous studies have showed that chronic alcohol exposure reduces GABA transmission (Hunt, 1983), other have demonstrated that chronic alcohol treatment increases GABA transmission in central amygdala (CeA) which is a brain region involved in anxiety and alcohol consumption behavior (Roberto et al., 2003; Koob, 2004; Roberto et al., 2004). We showed that chronic voluntarily alcohol drinking increased the levels of GABA in the brain tissues of female cHAP mice. A further study examining some brain structures specifically involved in the anxiety response, such as the amygdala may be necessary to establish a role of altered GABA levels in anxiety-like behaviors in this model. In addition, it may be desirable to treat these alcohol-exposed cHAP mice with GABA antagonists to determine whether GABA signaling is indeed involved in alcohol-induced anxiety.

CRF has been identified as a mediator of alcohol’s action on GABA signaling in the CeA as alcohol-enhanced GABA signaling was blocked in CeA neurons in CRF1 receptor knockout mice (Nie et al., 2004). CRF and CRFR1 receptors have been implied in alcohol consumption (Lodge and Lawrence, 2003; Hansson et al., 2006; Cippitelli et al., 2012; Lowery-Gionta et al., 2012). Chronic feeding of alcoholic liquid diet increased the mRNA level of CRF in the CeA (Läck et al., 2005). CRF signaling may also be involved in anxiety behavior as CRF administration caused anxiety (Dunn and Berridge, 1990), whereas CRFR1 antagonists or knock-out of CRFR1 reduced anxiety-like behavior (Timpl et al., 1998; Zorrilla et al., 2002; Müller et al., 2003; Henckens et al., 2016). Therefore, the increased CRF level in alcohol-exposed cHAP mice, may contribute to alcohol consumption and anxiety-like behaviors. However, as discussed above, it is desirable to examine the levels of CRF in some brain regions that are involved in the regulation of anxiety, such as the amygdala. Further experiments using CRFR1 antagonists or knock out of CFR/CRFR1 in this model are necessary to confirm its role.

The neurotrophin BDNF is affected by alcohol exposure and associated with alcohol drinking behaviors (McGough et al., 2004; Jeanblanc et al., 2009; Logrip et al., 2009; Tapocik et al., 2014; Stragier et al., 2015). BDNF expression appears to have an inverse correlation with alcohol intake. There were a number of studies showing decreased BDNF level promoted behavioral responses to alcohol whereas increased level of BDNF attenuated alcohol preference (McGough et al., 2004; Jeanblanc et al., 2009). However, the findings regarding the relationship between BDNF level and anxiety disorder are not consistent in either animal models (Chen et al., 2006; Govindarajan et al., 2006; Monteggia et al., 2007) or human studies (Maina et al., 2010; Molendijk et al., 2011; Wang et al., 2011). The increased BDNF level observed in the alcohol-exposed cHAP in this study, may account for the neurogenesis observed in the DG and SVZ (Figure 9). However, the impact of increased BDNF levels on anxiety in this study is unclear. Therefore, future studies using BDNF knockout mice or knockdown in this model will be desired to further determine the role of BDNF.

CREB is an alcohol-sensitive transcription factor (Uddin and Singh, 2007). Reduced levels of CREB and pCREB have been linked to the high anxiety and excessive alcohol intake in alcohol-preferring rats (Pandey et al., 2005). In addition, partial deletion of CREB caused alcohol-drinking and anxiety-like behaviors in mice (Pandey et al., 2004). We showed that the expression of CREB protein was decreased in the brain, particularly in the hippocampus and prefrontal cortex (PFC), the two brain regions that are involved in the control of stress-related behaviors (Russo and Nestler, 2013) and alcohol drinking behaviors (Koob and Roberts, 1999). A further study on the levels of CREB in other brain regions, such as the CeA and medial nuclei of amygdala (MeA), may be necessary to draw a conclusion. In addition, to determine whether the reduced CREB level contributes to alcohol-induced anxiety of in cHAP mice, we may infuse activators for CREB’s upstream protein kinase PKA, or Neuropeptide Y, one of the CREB-targeted genes, into the amygdala, hippocampus or prefrontal cortex in future study.

The female sex hormones and their receptors have been proposed to modulate anxiety in both humans (Maeng and Milad, 2015; Li and Graham, 2017) and rodents (Zimmerberg and Farley, 1993; Toufexis, 2007; Domonkos et al., 2017). We demonstrate that chronic voluntary alcohol drinking decreased G-protein-coupled estrogen receptor GPR30 in the brain while had little effect on estradiol/progesterone levels in the blood. It has been shown that administration of GPR30 agonist G-1 can decrease anxiety in female mice suggesting anxiolytic effects of GPR30 (Tian et al., 2013; Anchan et al., 2014; Liu et al., 2015). Future experiments using GPR30 agonist G-1 will be able to determine the role of GPR30 in alcohol-induced anxiety in female cHAP mice.

### Chronic Voluntary Alcohol Drinking Induced Oxidative Stress, ER Stress, Neuroinflammation and Neurodegeneration.

Oxidative stress, ER stress, neuroinflammation have been proposed as potential mechanisms for alcohol-induced neurodegeneration. For example, oxidative stress is associated with alcohol-induced neurodegeneration *in vitro* and *in vivo* (Chen and Luo, 2010; Hernández et al., 2016). There have been many studies showing a correlation between oxidative stress and alcohol dependence (Thome et al., 1997; Das et al., 2007; Haorah et al., 2008; Galicia-Moreno and Gutierrez-Reyes, 2014; Muñiz Hernandez et al., 2014). The link between oxidative stress and anxiety has been established in human patients or animal models and oxidative stress has been suggested to play a causative role in anxiety (Bouayed et al., 2009; Hovatta et al., 2010). Similar to our previous findings in male cHAP mice (Xu et al., 2019), chronic voluntary alcohol drinking also caused oxidative stress in female cHAP mice. Therefore, alcohol-induced oxidative stress in cHAP mice is not gender-specific. To further determine whether oxidative stress is involved in the alcohol dependence and anxiety in this model, future studies could use antioxidants or free radical scavengers in alcohol-drinking cHAP mice.

ER stress is another potential mechanism for alcohol-induced brain damage in both developing and adult brains (Yang and Luo, 2015). Similar to our previous findings in male cHAP mice (Xu et al., 2019), chronic voluntary alcohol drinking caused ER stress in female cHAP mice. There is little information regarding the role of ER stress in alcohol-drinking behaviors. However, ER stress may play a critical role in stress-related behaviors as treatment of 4-phenylbutyrate (4-PBA), a known ER stress inhibitor, alleviated anxiety-like behaviors in a mouse model for diffuse axonal injury (Huang et al., 2019). The administration of 4-PBA, and edaravone, a free radical scavenger, improved chronic restraint stress (CRS)-induced anxiety-like behaviors in mice which were accompanied with inhibition of oxidative stress, neuroinflammation and ER stress (Jangra et al., 2017).

In addition, chronic alcohol voluntary drinking caused caspase-3 activation in the brain, particularly the PFC and hippocampus, indicative of apoptosis. The PFC and hippocampus are parts of the brain reward circuitry that is vulnerable to alcohol exposure (Sunkesula et al., 2008; Wang et al., 2018; Xu et al., 2019). Again, these findings are consistent with our previous results obtained from male cHAP mice (Xu et al., 2019).

The alcohol-induced neurodegeneration in both adult and developing brain is often accompanied by neuroinflammation and gliosis (Alfonso-Loeches et al., 2013; Pascual et al., 2014; Tajuddin et al., 2014; Zhang and Luo, 2019). We showed that chronic alcohol exposure induced neuroinflammation which was demonstrated by the increased expression of proinflammatory TNFα, MCP-1 and its receptor CCR2 but not IL-6; alcohol also increased gliosis which was indicated by increased GFAP-labeled astrocytes (He and Crews, 2008; Sullivan and Zahr, 2008). The alcohol-induced neuroinflammation may also contribute to the anxiety-like behavior as higher basal levels of TNFα and MCP-1 were observed in patients with anxiety (Vogelzangs et al., 2016). Treatment TNFα inhibitors have been shown to improve the symptoms of anxiety in patients (Ertenli et al., 2012) and animals (Haji et al., 2012).

### Chronic Voluntary Alcohol Drinking Reduced Thiamine in the Blood

Thiamine deficiency (TD) has long been associated with alcoholism (Leevy and Baker, 1968; Woodhill and Nobile, 1972; Butterworth, 1995) and has been implicated as a key factor for alcohol-induced brain damages (Martin et al., 2003; Liu et al., 2017). Our recent publication indicated thiamine deficiency can cause anxiety-like behaviors (Li et al., 2020). Xu et al. showed chronic voluntary alcohol drinking caused a significant decrease in the levels of thiamine in the brain but not the blood of male cHAP mice (Xu et al., 2019). In the present study, alcohol drinking significantly reduced thiamine concentration in the blood of female cHAP (Table 1), and decreased the expression of thiamine transporters (SLC19A2, SLC19A3, and OCT1) in female cHAP mice (Figure 7). Our previous study showed that alcohol exposure down-regulated the expression of SLC19A3, but not SLC19A2 and OCT1 in male cHAP mice (Xu et al., 2019). Due to some technical problems, we were unable to detect and quantify thiamine in the brain of female cHAP mice. Further effort is needed to evaluate thiamine levels in the brain of female cHAP mice. The reduced levels of thiamine either in the brain and blood may contribute to anxiety-like behaviors.

### Chronic Voluntary Alcohol Drinking Stimulated Neurogenesis in the Brain

Depending on the paradigms of alcohol exposure and experimental models employed, alcohol exposure could either inhibit or stimulate neurogenesis (Nixon and Crews, 2002; He et al., 2005; Ieraci and Herrera, 2007; Klintsova et al., 2007; Morris et al., 2010; Hamilton et al., 2011). In the current study, we used multiple markers, such as DCX, BrdU and Ki67 to monitor neurogenesis and demonstrated that chronic voluntary alcohol drinking increased the neurogenesis in the DG and SVZ of female cHAP mice. This findings is consistent with the result obtained from male cHAP mice (Xu et al., 2019). Therefore, the effect of chronic voluntary alcohol drinking on the neurogenesis in cHAP mice is not gender specific. The enhanced neurogenesis observed in these two regions may result from the increased BDNF expression and/or compensatory response to alcohol-induced neuronal damage.

In summary, chronic voluntary alcohol drinking caused anxiety-like behaviors, and altered the expression of several neurotransmitters and neurotrophic factors associated with the regulation of anxiety in female cHAP mice. In comparison with our previous study in male cHAP mice, the current results indicate that alcohol-induced damage and neurochemical changes to the brain, such as oxidative stress, ER stress, neurodegeneration and neurogenesis, are similar between male and female mice and not gender-specific. It appears that the effect of alcohol exposure on thiamine concentrations in the blood and the expression of thiamine transporters is different between male and female cHAP mice. Further studies are needed to investigate the underlying mechanisms.

## Data Availability

The original data that support the findings of this study are available from the corresponding author upon reasonable request.

## Abbreviations

AUD, alcohol use disorders; BrdU, Bromodeoxyuridine; BDNF, Brain-derived neurotrophic factor; CCR2, C-C chemokine receptor 2; cHAP mice, Crossed High Alcohol Preferring mice; CNS, central nervous system; CREB, cAMP response element-binding protein; CRF, Corticotropin-releasing factor; DCX, doublecortin; DG, dentate gyrus of the hippocampus; ER, endoplasmic reticulum; GPR30, G protein-coupled estrogen receptor 1; GFAP, Glial fibrillary acidic protein; IHC, immunohistochemistry; Iba-1, Ionized calcium binding adaptor molecule 1; IL-6, Interleukin-6; MANF, Mesencephalic astrocyte-derived neurotrophic factor; MCP-1, monocyte chemoattractant protein-1; OCT1, Organic cation transporter 1; PBS, phosphate buffered saline; SGZ, subgranular zone; SLC19A2, Solute Carrier Family 19 Member 2 (Thiamine transporter 1); SLC19A3, Solute Carrier Family 19 Member 2 (Thiamine transporter 2); SVZ, subventricular zone; TNFα, tumor necrosis factor alpha.
